# The Independence of the Fœtal Circulation

**Published:** 1842-10-08

**Authors:** 


*The Independence of the Fœtal Circulation.*—There is a memoir on this subject by Dr. Villeneuve, professor in the School of Medicine at Marseilles, which rather confirms the already received opinions on this subject than proposes anything new. The conclusions he arrives at are chiefly these:—1. There is no anastomotic communication between the fœtal and maternal circulation in the human being, as in the generality of quadrupeds. This absence of communication is proved by the existence of an elementary apparatus for circulation in the embryo, before the formation of the placenta; by the plethoric condition in which the fœtus is often found in cases where the mother has suffered severely from uterine hæmorrhage, as in placenta prævia when the placenta has not been injured, and by the continuance of the placento-fœtal circulation without hæmorrhage in an ovum expelled entire. 2. The death of the fœtus is only due to the want of oxygenation of the blood, and may therefore be prevented by accelerating delivery. It is never due to anemia, except when the umbilical vessels of the placenta are torn. 3. The death of the mother may be occasioned by the separation of a small portion of the placenta, which proves it to be rather owing to venous than arterial hæmorrhage, inasmuch as the utero-placental veins have numerous communications with one another, both in the placenta and in the uterus, whereas the arteries present few if any anastomoses. 4. Ergot of rye is injurious both to mother and child in hæmorrhage from placenta prævia, because it determines the constriction of the uterus as much or more at the lower part than at the upper, without producing dilatation of the external orifice, and by the contractions which it excites it expels from the uterus the blood which the mother has need to preserve. 5. The plug, if employed at the proper period, is the best treatment for such cases. It determines more certainly than ergot the proper contraction of the uterus. If applied too late, it is ineffectual in controlling the hæmorrhage. 6. It is very bad practice to pass the hand through the placenta in the endeavour to effect the artificial delivery of the child. It is always easy to separate the placenta at the point from which the hæmorrhage proceeds. By the former method the death of the child is most decidedly accelerated, in consequence of the rupture of the umbilical vessels. 7. We may hope to find the child alive, even after the death of the mother, especially in cases where severe hæmorrhage has quickly proved fatal to her. Hence the obligation to practise the Cæsarean operation on women who die during labour.—*London Med. Gaz.* Aug. 26, 1842. *From Gazette Médicale.*

